# Delayed vaccination and its predictors among children under 2 years in India: Insights from the national family health survey–4

**DOI:** 10.1016/j.vaccine.2019.03.039

**Published:** 2019-04-17

**Authors:** Tarun Shankar Choudhary, N. Samarasimha Reddy, Aditi Apte, Bireshwar Sinha, Sudipto Roy, Nayana P. Nair, Kulandaipalayam Natarajan Sindhu, Rutuja Patil, Ravi Prakash Upadhyay, Ranadip Chowdhury

**Affiliations:** a Research Scientist and PRERNA Young Investigator, Centre for Health Research and Development, Society for Applied Studies, New Delhi, India; b PRERNA Young Investigator, Christian Medical College, Vellore, India; c PRERNA Young Investigator, KEM Hospital Research Centre, Pune, India

**Keywords:** Timeliness, Delayed Vaccination, Measles, DPT, Child health, Epidemiology

## Abstract

**Objective:**

Delayed vaccination increases the susceptibility window for vaccine preventable diseases. Our analysis estimates the proportion of children between 10 and 23 months of age with delayed vaccination in India and the associated socio-demographic, maternal and child related factors.

**Methods:**

We used individual level data from the National Family and Health Survey 4, conducted in 2015–2016. The primary outcome of the study was delayed vaccination for BCG, DPT- 1st dose and Measles. Delayed vaccination for each vaccine was defined as administration of the vaccine dose after 28 days of the minimum recommended age, as per the national immunization schedule in India. We estimated the proportion of children with delayed vaccination for each vaccine and used multivariable logistic regression to explore associated factors.

**Findings:**

In the current analysis, 23.1%, 29.3% and 34.8% of children aged 10 to 23 months had delayed vaccination for BCG, DPT-1st dose and Measles respectively. Children from Muslim families (aOR 1.36 for BCG; aOR 1.45 for DPT-1; aOR 1.26 for Measles); birth weight < 2000 g (aOR 2.33 for BCG; aOR 1.53 for DPT-1; aOR 1.36 for Measles) had higher odds of delayed vaccination. Lower maternal education and belonging to a family from lower wealth quintile had higher odds of delayed vaccination. Children of mothers who had tetanus toxoid immunization during pregnancy had lower odds of delayed vaccination (aOR 0.69 for BCG; aOR 0.76 for DPT-1; aOR 0.78 for Measles).

**Conclusion:**

The proportion of children with delayed vaccination is high in India. Vaccine timeliness should be a core indicator of the immunization program with greater focus on groups with higher chances of delayed vaccination i.e. home birth, low birth weight new-borns, poorer households, children of mothers with lower education and children from Muslim families.

## 1 Introduction

Globally, around 29% of under-five deaths were due to vaccine preventable diseases (VPD) in 2017 [1]. Despite a substantial decline in the under-five mortality, India accounted for the highest number of under-five deaths globally in 2016 [2]. VPDs like diarrhoea, pneumonia and measles were among the leading causes of under-five deaths in India resulting in about one-fourth of all under-five deaths between 2000 and 2015 [3]. India has high burden of Pertussis, Diphtheria, Japanese Encephalitis and Measles despite a national immunization program in place since the last five-decades [3], [4], [5]. Complete and timely vaccination can potentially reduce childhood mortality [6].

The Expanded Programme of Immunization was introduced in India in 1978. It has been scaled up considerably in terms of population covered as well as the number of targeted pathogens [7]. The proportion of children fully immunized at one year of age (defined as receiving BCG, Measles, and 3 doses each of oral polio and Diphtheria, Pertussis, Tetanus toxoid) has increased from 44% in 2005–6 as per National Family Health Survey (NFHS)-3 to 62% in 2015–16 (NFHS-4) [8], [9]. The Government of India launched “Mission Indradhanush” in December 2014 to increase vaccination coverage for under-five children and pregnant women and has now launched Intensified Mission Indradhanush (IMI) to achieve full vaccination for > 90% of potential beneficiaries by December 2018 [10], [11]. New vaccines (pentavalent vaccine, oral rotavirus vaccine, injectable polio vaccine and pneumococcal vaccine) have been added to the Universal Immunisation Program (UIP) as detailed in Supplementary Table 1 [7]. However, vaccine timeliness, although an important indicator of the programme’s quality, has been a relatively neglected aspect of programme performance.

The recommended age for vaccination is based on two factors; the earliest age at which the immunity afforded by placental transfer of maternal antibodies at birth wanes, thereby making the infants susceptible to pathogens and the earliest age at which safety and efficacy of the vaccine has been demonstrated [12]. Delay in vaccination increases the susceptibility window for developing VPDs at individual level and reduces herd immunity at population level [6], [12], [13]. Evidence from previous studies have demonstrated that delayed vaccination may increase the risk of Pertussis, Measles and *Haemophilus influenzae B* infections up to 6 folds and lead to outbreaks [14], [15], [16], [17]. In case of certain vaccines with fixed upper age limit (e.g. rotavirus) delayed vaccination leads to reduced coverage for the vaccine [18].

The available literature on vaccine timeliness and its associated factors is limited in India [19], [20], [21]. The primary objective of the current analysis was to estimate the proportion of children aged 10 to 23 months with delayed vaccination for BCG, DPT-1st dose and Measles at national and subnational level in India using the recent NFHS-4 data. We also examined the association of delayed vaccination with socio-demographic, maternal and child related factors to identify population sub-groups at higher risk of delayed vaccination.

## 2 Methodology

### 2.1 Data source

This analysis was based on individual level data from the 4th round of the NFHS, a nationally representative cross-sectional survey. It provides reliable estimates on fertility, mortality, reproduction, child health and other demographic indicators at national, state and district level [8]. Around 628,900 households in 29 states and 7 union territories in India were interviewed for NFHS-4, with a response rate of 98%. A two-stage stratified sampling design with villages in rural areas and Census Enumeration Blocks (CEBs) in urban areas, forming the primary sampling units (PSU), was adopted during the first stage. Within each PSU, the households were selected using systematic random sampling in the second stage. Clinical, anthropometric and biochemical measurements for men, women and children were done. Detailed description of the sampling design and instruments used in the survey have been provided elsewhere [8].

**Fig. 1:**
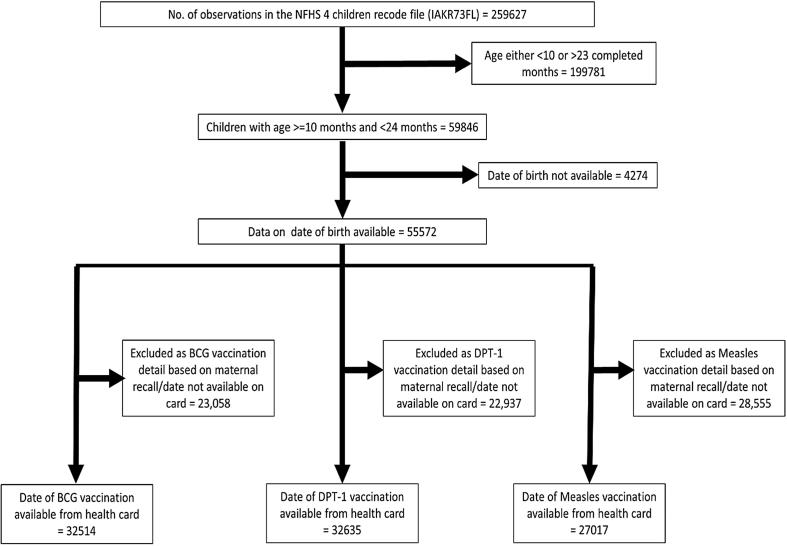
The detail of the case records from the child recode file used for the current analysis with the reasons for exclusion (NFHS 4, 2015–16).

We used the children recode file (IAKR73FL.dta), available from the Demography and Health Survey (DHS) program website, for this analysis [22]. Information related to the antenatal care and postnatal care for the respective pregnancy, immunization status of the child, along with data for maternal and household characteristics of the child was included in the recode file. Information on the child's vaccination status in NFHS-4 was based either on the Mother and Child Protection card (MCP)/Health card or mother's recall. In the current analysis, we have only included children for whom date of vaccination for a given vaccine dose was available on the MCP/Health card. Children for whom data for immunisation was based on maternal recall or the date of vaccination was not available on the health card were excluded from the analysis (details in Fig. 1). Ethical clearance was not needed as the analysis used secondary data available in the public domain. The guidelines for data use as required by the DHS program were strictly followed.

### 2.2 Definition of primary outcome

The primary outcome of the study was delayed vaccination for BCG, DPT- 1st dose and Measles. Delayed vaccination for each vaccine was defined as administration of the vaccine dose after 28 days of the minimum recommended age, as per the national immunization schedule in India (Supplementary Table 1). Date of birth and age at vaccination (for individual vaccine dose) was calculated in century day code format. Vaccination was categorised as delayed if given on day 29 or later for BCG, 71 days or later (after 10 completed weeks) for DPT-1st dose and 303 days or later (after 10 completed months) for Measles vaccine (Supplementary Box 1).

### 2.3 Statistical analysis

Proportion of children with delayed vaccination was estimated for BCG, DPT-1st dose, and Measles vaccination at national level and for individual states and union territories. Median age at vaccination for BCG, DPT-1st dose and Measles vaccine was calculated. Univariate analysis was done to examine the association between socio-demographic, maternal, antenatal and child characteristics and delayed vaccination. Socio-demographic variables included religion, caste, place of residence and wealth quintile. Maternal and antenatal variables included maternal education, age at birth of 1st child, maternal tetanus immunisation during pregnancy and financial assistance for delivery. Child level variables included gender, place of delivery, birth weight and birth order. Details of the variables and the sub-categories are available in Supplementary Table 2.

Multivariable logistic regression models were built to examine the association between delayed vaccination for BCG, DPT- 1st dose and Measles, individually. Variables with a *p*-value < 0.25 on univariate analysis and those of known clinical or contextual importance were included in the multivariable logistic regression analysis [23]. Stepwise backward elimination based on design-based Wald test was used to finalise the model. A p-value of < 0.05 was considered statistically significant. STATA version 15.1 (StataCorp LLC, College Station, TX, USA) was used for all analysis and adjustment for sampling weight, clustering and strata was done using *svyset* command.

## 3 Results

We analysed data of 32,514 children for BCG, 32,635 children for DPT-1st dose and 27,017 children for delayed Measles vaccination (Fig. 1). Details of the background characteristics are provided in Table 1.

**Table 1:** Background characteristics for delayed BCG, delayed DPT-1st dose and delayed Measles vaccination among children 10 to 23 months of age in India (NFHS 4, 2015–16).

	BCG		DPT-1		BCG	
Variables	No Delay	Delay	No Delay	Delay	No Delay	Delay
	N (%)	N (%)	N (%)	N (%)	N (%)	N (%)
**Socio-demographic characteristics**						
*Religion (total)*						
Hindu	17,900 (79.82)	5677 (76.12)	16840 (80.46)	6911 (76.33)	12984 (80.32)	6712 (77.84)
Muslim	3495 (14.19)	1377 (19.35)	3068 (13.45)	1713 (19.22)	2287 (13.1)	1521 (17.31)
Others*	2858 (6)	1207 (4.53)	3042 (6.1)	1061 (4.45)	2396 (6.58)	1117 (4.86)

*Caste (total)*						
Scheduled caste	4778 (23.25)	1499 (22.42)	4350 (22.93)	1974 (23.55)	3259 (22.18)	1916 (24.11)
Scheduled tribe	4186 (9.62)	1673 (10.35)	4200 (9.59)	1685 (10.2)	3208 (9.35)	1602 (9.33)
Other backward classes	9632 (45.61)	3163 (48.21)	8878 (45.76)	4018 (47.84)	6849 (45.36)	3714 (46.44)
Others**	4484 (21.52)	1507 (19.02)	4390 (21.73)	1575 (18.4)	3463 (23.11)	1677 (20.12)

*Wealth index (total)*						
Highest	4495 (18.7)	878 (11.43)	4360 (19.53)	992 (10.24)	3514 (20.4)	1333 (14.82)
Fourth	4843 (21.35)	1371 (16.69)	4754 (22.35)	1476 (15.36)	3686 (22.32)	1680 (19.18)
Middle	5242 (21.52)	1809 (20.56)	4974 (21.37)	2081 (20.75)	3871 (21.46)	2019 (20.8)
Second	5105 (20.32)	2191 (25.07)	4800 (19.6)	2549 (26.07)	3613 (19.19)	2216 (23.1)
Lowest	4568 (18.1)	2012 (26.26)	4062 (17.15)	2587 (27.58)	2983 (16.63)	2102 (22.1)

*Place of residence (total)*						
Urban	6483 (31.01)	1878 (23.93)	6222 (31.27)	2087 (23.45)	4769 (31.21)	2370 (29.29)
Rural	17770 (68.99)	6383 (76.07)	16728 (68.73)	7598 (76.55)	12898 (68.79)	6980 (70.71)

**Maternal and antenatal characteristics**						
*Maternal education (total)*						
Higher	3150 (13.98)	709 (8.73)	3175 (15.1)	679 (6.75)	2535 (15.71)	963 (11.14)
Secondary	12828 (53.75)	4082 (47.12)	12333 (53.77)	4618 (47.54)	9632 (54.66)	4723 (50.04)
Primary	3116 (12.68)	1286 (15.44)	2915 (12.55)	1522 (15.49)	2171 (12.19)	1385 (14.65)
None	5159 (19.59)	2184 (28.71)	4527 (18.57)	2866 (30.23)	3329 (17.43)	2279 (24.17)

*Maternal age at first child birth (total)*						
Less than 19	4181 (19.11)	1714 (21.98)	3856 (18.28)	2060 (23.48)	2884 (17.81)	1838 (21.95)
19 – 30	19537 (79.14)	6362 (76.81)	18515 (79.83)	7477 (75.57)	14325 (80.17)	7322 (76.83)
31 and above	535 (1.75)	185 (1.21)	579 (1.89)	148 (0.95)	458 (2.02)	190 (1.22)

*Antenatal visits to health facility (total)*						
No ANC visits	2242 (9.78)	1245 (16.19)	2098 (9.51)	1440 (16.02)	1524 (8.87)	1076 (12.41)
1–3 visits	7412 (28.73)	3049 (38.01)	6822 (27.86)	3700 (38.2)	5238 (27.88)	3174 (33.05)
4–8 visits	9636 (41.06)	2837 (34.76)	9414 (42.12)	3113 (33.55)	7306 (42.14)	3372 (38.03)
>8 visits	3759 (20.43)	690 (11.04)	3548 (20.51)	858 (12.24)	2777 (21.11)	1155 (16.51)

*Financial assistance at the time of delivery (total)*						
Yes	10928 (45.6)	2519 (40.39)	9746 (43.68)	3785 (48.01)	7433 (43.13)	3827 (45.92)
No	9960 (54.4)	3050 (59.61)	9589 (56.32)	3381 (51.99)	7466 (56.87)	3554 (54.08)

*Tetanus injections before birth (total)*						
No tetanus injections	1121 (5.11)	502 (6.7)	1052 (5.23)	550 (6.18)	766 (4.88)	455 (5.89)
At least one tetanus injection	22032 (94.89)	7351 (93.3)	20935 (94.77)	8594 (93.82)	16149 (95.12)	8365 (94.11)

**Child characteristics**						
*Gender of the child (total)*						
Male	12643 (51.98)	4301 (52.28)	11981 (51.98)	5007 (51.51)	9214 (51.6)	4832 (52.29)
Female	11610 (48.02)	3960 (47.72)	10969 (48.02)	4678 (48.49)	8453 (48.4)	4518 (47.71)

*Place of delivery (total)*						
Home births (total)	2438 (8.83)	2402 (26.34)	2823 (10.27)	2104 (20.12)	2151 (10.5)	1538 (14.49)
Private health facility	5570 (28.55)	2006 (28.9)	5658 (30.39)	1947 (24.34)	4420 (31.17)	2142 (27.83)
Public health facility	16245 (62.62)	3853 (44.76)	14469 (59.33)	5634 (55.54)	11096 (58.34)	5670 (57.69)

*Birth weight (total)*						
<2000 g	728 (3.17)	362 (7.23)	678 (3.31)	414 (5.68)	566 (3.56)	369 (4.69)
2000–2499 g	2760 (13.13)	700 (12.56)	2453 (12.82)	1017 (13.4)	1924 (13.06)	998 (12.91)
≥2500 g	18338 (83.7)	4980 (80.21)	17250 (83.87)	6104 (80.92)	13241 (83.38)	6483 (82.4)

*Birth order (total)*						
1st	10107 (42.7)	2949 (36.48)	9667 (43.41)	3460 (36.16)	7699 (45.06)	3562 (38.5)
2nd	8069 (34.73)	2544 (32.08)	7610 (34.8)	2991 (31.78)	5803 (34.42)	3046 (34.21)
3rd	3478 (13.56)	1397 (15.93)	3228 (13.11)	1675 (16.91)	2384 (12.53)	1500 (15.11)
4 or more	2599 (9)	1371 (15.51)	2445 (8.69)	1559 (15.15)	1781 (7.99)	1242 (12.17)

* Others (Christians, Sikh, Buddhist/neo-Buddhist, Jain, Jewish, Parsi/Zoroastrian, no religion, Other (not defined)).

** Others (do not belong to Scheduled caste/Tribe and other backward castes).

### 3.1 Delayed vaccination

**Fig. 2:**
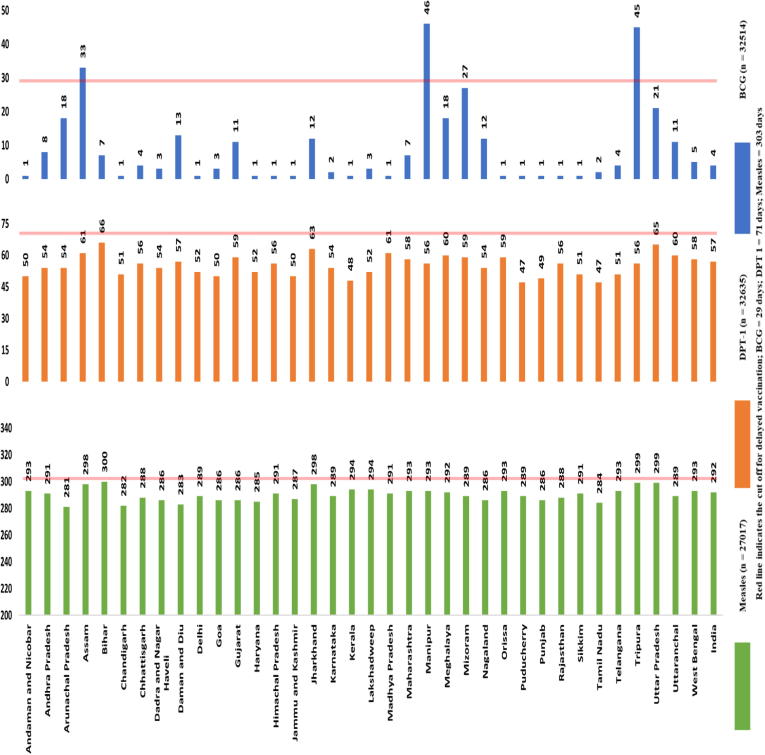
The median age at vaccination (in days) for BCG, DPT-1 and Measles at State and National level in India for children 10 to 23 months of age (NFHS 4, 2015–16).

**Fig. 3:**
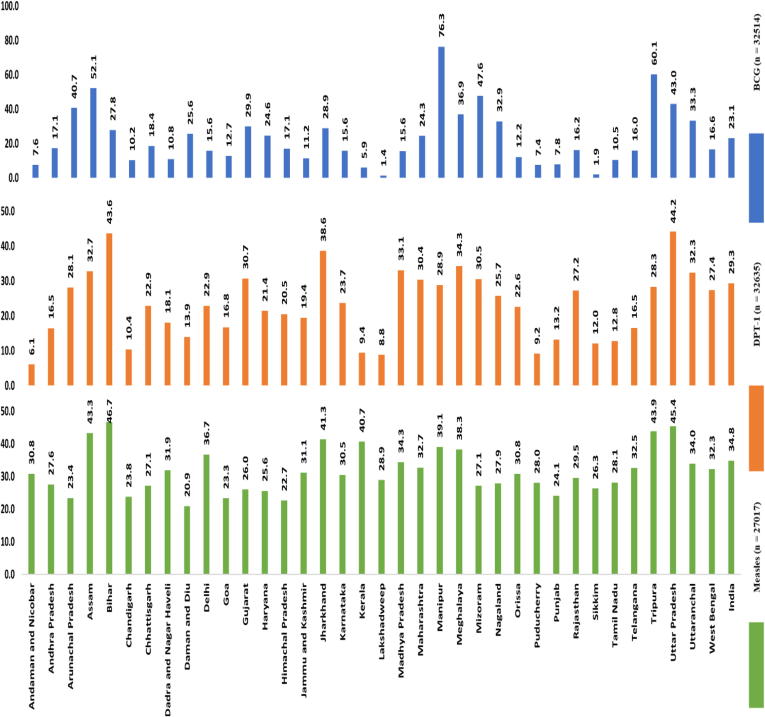
Figure showing the proportion of children aged 10 to 23 months of age with delayed vaccination for BCG, DPT-1 and Measles at state and national level in India (NFHS 4, 2015–16).

The median age for BCG, DPT–1st dose and Measles vaccination was four days, 57 days (8 weeks and 1 day) and 292 days (9 months and 18 days) respectively (Fig. 2). Nationally, 23.1%, 29.3% and 34.8% of children aged 10 to 23 months had delayed vaccination for BCG, DPT-1st dose and Measles respectively. The proportion of children with delayed vaccination across different states and union territories in India ranged from 1.4% to 76.3% for BCG, 6.14% to 44.2% for DPT-1st dose and 20.9% to 46.7% for Measles (Fig. 3).

### 3.2 Factors associated with delayed vaccination

Table 2, Table 3, Table 4 shows the results from the multivariable logistic regression analysis for delayed BCG, delayed DPT-1st dose and delayed Measles vaccination respectively. Children from Muslim families had higher odds of delayed BCG (aOR 1.36, 95% CI 1.17, 1.57), delayed DPT-1st dose (aOR 1.45, 95% CI 1.27, 1.65) and delayed Measles (aOR 1.26, 95% CI 1.09, 1.45) vaccination. Those with a birth weight < 2000 g had higher odds of delayed BCG (aOR 2.33, 95% CI 1.89, 2.89), delayed DPT-1st dose (aOR 1.53, 95% CI 1.26, 1.86) and delayed Measles (aOR 1.36, 95% CI 1.11, 1.67) vaccination. A dose-response like relationship was seen between the wealth index of child family and educational status of child’s mother. Compared to children from highest wealth quintile those from lower wealth quintiles had higher chances of delayed vaccination for BCG, DPT-1st dose and Measles in the adjusted analysis. Children of mothers with higher education (≥12 years of schooling) had lower odds of delayed vaccination for BCG, DPT-1st dose and Measles as compared to children of mothers with lower education. Maternal tetanus immunisation during pregnancy was associated with lower odds of delayed BCG (aOR 0.69, 95% CI 0.56, 0.85), delayed DPT-1st dose (aOR 0.76, 95% CI 0.62, 0.94) and delayed Measles vaccination (aOR 0.78, 95% CI 0.63, 0.97). Maternal age of <19 years at first childbirth was associated with higher odds of delayed DPT-1st dose (aOR 1.19, 95% CI 1.07, 1.32) and delayed Measles vaccination (aOR 1.18, 95% CI 1.06, 1.32). Children from rural areas had higher odds of delayed Measles vaccination (aOR 0.87, 95% CI 0.77, 0.98) but not for BCG or DPT-1st dose. Home births and births in a private health facility were associated with higher odds of delayed BCG (aOR 2.67, 95% CI 2.25, 3.17 and aOR 1.77, 95% CI 1.57, 1.99) and delayed DPT-1st dose vaccination (aOR 1.41, 95% CI 1.20, 1.64 and aOR 1.18, 95% CI 1.06, 1.31), respectively. Higher birth order of the child was found to have higher odds of delayed Measles vaccination. Gender of the child didn’t have a significant association with delayed BCG, delayed DPT-1st dose and delayed Measles vaccination.

**Table 2:** Logistic regression model showing predictors for delayed BCG vaccination among infants 10–23 months of age in India (NFHS 4, 2015–16).

Socio-demographic characteristics	BCG n = 26611 (weighted)		
	Unadjusted odds ratio (95%CI)	Adjusted Odds ratio (95% CI)	p value
*Religion*			
Hindu	Reference	Reference	–
Muslim	1.43 (1.29, 1.59)	1.36 (1.17, 1.57)	0.000
Others*	0.79 (0.69, 0.94)	1.03 (0.79, 1.35)	0.810

*Wealth index*			
Highest	Reference	Reference	–
Fourth	1.28 (1.1, 1.49)	1.28 (1.05, 1.56)	0.016
Middle	1.56 (1.35, 1.81)	1.54 (1.24, 1.91)	0.000
Second	2.02 (1.75, 2.33)	1.77 (1.41, 2.23)	0.000
Lowest	2.37 (2.06, 2.73)	1.95 (1.51, 2.51)	0.000

*Place of residence*			
Urban	Reference	Reference	–
Rural	1.43 (1.3, 1.57)	1.08 (0.95, 1.22)	0.268

**Maternal and antenatal characteristics**			
*Maternal education*			
Higher	Reference	Reference	–
Secondary	1.4 (1.23, 1.61)	1.23 (1.05, 1.46)	0.013
Primary	1.95 (1.67, 2.28)	1.33 (1.08, 1.64)	0.008
None	2.35 (2.04, 2.71)	1.37 (1.09, 1.71)	0.007

*Mother received at least one tetanus toxoid injection during pregnancy*			
No		Reference	–
Yes	0.75 (0.64, 0.88)	0.69 (0.56, 0.85)	0.000

**Child characteristics**			
*Gender of the child*			
Male	Reference	Reference	–
Female	0.99 (0.92, 1.06)	0.96 (0.88, 1.06)	0.434

*Place of delivery*			
Public health facility	Reference	Reference	–
Private health facility	1.42 (1.3, 1.55)	1.77 (1.57, 1.99)	0.000
Home births	4.17 (3.76, 4.63)	2.67 (2.25, 3.17)	0.000

*Birth weight*			
<2000 g	2.38 (1.98, 2.87)	2.33 (1.89, 2.89)	0.000
2000–2499 g	1 (0.88, 1.14)	1.03 (0.90, 1.18)	0.669
≥ 2500 g	Reference	Reference	–

* Others (Christians, Sikh, Buddhist/neo-Buddhist, Jain, Jewish, Parsi/Zoroastrian, no religion, Other (not defined)).

**Table 3:** Logistic regression model showing predictors for delayed DPT1 vaccination among infants 10 to 23 months of age in India (NFHS 4, 2015–16).

	DPT1 n = 26649 (weighted)		
	Unadjusted odds ratio (95%CI)	Adjusted Odds ratio (95% CI)	p value
**Socio-demographic characteristics**			
*Religion*			
Hindu	Reference	Reference	–
Muslim	1.51 (1.36, 1.67)	1.45 (1.27, 1.65)	0.000
Others*	0.77 (0.65, 0.9)	1.18 (0.94, 1.49)	0.152

*Wealth index*			
Highest	Reference	Reference	–
Fourth	1.31 (1.14, 1.51)	1.17 (0.98, 1.39)	0.082
Middle	1.85 (1.62, 2.12)	1.55 (1.30, 1.85)	0.000
Second	2.54 (2.24, 2.88)	1.76 (1.46, 2.12)	0.000
Lowest	3.07 (2.7, 3.49)	1.77 (1.44, 2.17)	0.000

*Place of residence*			
Urban	Reference	Reference	–
Rural	1.48 (1.35, 1.63)	0.98 (0.87, 1.10)	0.731

**Maternal and antenatal characteristics**			
*Maternal education*			
Higher	Reference	Reference	–
Secondary	1.98 (1.73, 2.26)	1.51 (1.29, 1.76)	0.000
Primary	2.76 (2.38, 3.21)	1.62 (1.31, 1.91)	0.000
None	3.64 (3.17, 4.19)	1.81 (1.50, 2.19)	0.000

*Maternal age at first child birth*			
Less than 19	1.36 (1.24, 1.48)	1.19 (1.07, 1.32)	0.001
19–30	Reference	Reference	–
Above 30	0.53 (0.4, 0.72)	0.68 (0.48, 0.95)	0.023

*Mother received at least one tetanus toxoid injection during pregnancy*			
No		Reference	–
Yes	0.84 (0.71, 0.99)	0.76 (0.62, 0.94)	0.010

**Child characteristics**			
*Gender of the child*			
Male	Reference	Reference	–
Female	1.02 (0.95, 1.09)	0.99 (0.91, 1.07)	0.745

*Place of delivery*			
Public health facility	Reference	Reference	–
Private health facility	0.86 (0.79, 0.93)	1.18 (1.06, 1.31)	0.002
Home births	2.09 (1.91, 2.3)	1.41 (1.20, 1.64)	0.000

*Birth weight*			
<2000 g	1.78 (1.49, 2.13)	1.53 (1.26, 1.86)	0.000
2000–2499 g	1.08 (0.97, 1.21)	1.07 (0.95, 1.20)	0.267
≥ 2500 g	Reference	Reference	–

* Others (Christians, Sikh, Buddhist/neo-Buddhist, Jain, Jewish, Parsi/Zoroastrian, no religion, Other (not defined)).

**Table 4:** Logistic regression model showing predictors for delayed measles vaccination among infants 10 to 23 months of age in India (NFHS 4, 2015–16).

	Measles n = 22,693 (weighted)		
	Unadjusted odds ratio (95%CI)	Adjusted Odds ratio (95% CI)	p value
**Socio-demographic characteristics**			
*Religion*			
Hindu	Reference	Reference	–
Muslim	1.36 (1.21, 1.53)	1.26 (1.09, 1.45)	0.002
Others*	0.76 (0.66, 0.88)	0.88 (0.73, 1.06)	0.186

*Wealth index*			
Highest	Reference	Reference	–
Fourth	1.18 (1.02, 1.37)	1.14 (0.97, 1.34)	0.124
Middle	1.33 (1.17, 1.52)	1.22 (1.03, 1.44)	0.020
Second	1.66 (1.45, 1.89)	1.37 (1.14, 1.64)	0.001
Lowest	1.83 (1.61, 2.08)	1.30 (1.08, 1.58)	0.007

*Place of residence*			
Urban	Reference	Reference	–
Rural	1.1 (1.00, 1.20)	0.87 (0.77, 0.98)	0.026

**Maternal and antenatal characteristics**			
*Maternal education*			
Higher	Reference	Reference	–
Secondary	1.29 (1.13, 1.47)	1.20 (1.04, 1.40)	0.016
Primary	1.7 (1.45, 1.98)	1.42 (1.18, 1.72)	0.000
None	1.96 (1.70, 2.25)	1.41 (1.70, 1.70)	0.000

*Maternal age at first child birth*			
less than 19	1.29 (1.17, 1.42)	1.18 (1.06, 1.32)	0.003
19–30	Reference	Reference	–
Above 30	0.63 (0.48, 0.82)	0.72 (0.55, 0.96)	0.023

*Mother received at least on tetanus toxoid injection during pregnancy*			
No		Reference	–
Yes	0.82 (0.68, 0.98)	0.78 (0.63, 0.97)	0.025

**Child characteristics**			
*Gender of the child*			
Male	Reference	Reference	–
Female	0.97 (0.91, 1.05)	0.96 (0.89, 1.04)	0.370

*Birth weight*			
<2000 g	1.33 (1.1, 1.61)	1.36 (1.11, 1.67)	0.003
2000–2499 g	1 (0.89, 1.13)	0.98 (0.86,1.11)	0.706
≥ 2500 g	Reference	Reference	–

*Birth order*			
1	Reference	Reference	–
2	1.16 (1.06, 1.27)	1.14 (1.03, 1.26)	0.012
3	1.41 (1.26, 1.58)	1.17 (1.02, 1.33)	0.021
4 or more	1.78 (1.58, 2.01)	1.27 (1.09, 1.48)	0.002

* Others (Christians, Sikh, Buddhist/neo-Buddhist, Jain, Jewish, Parsi/Zoroastrian, no religion, Other (not defined)).

## 4 Discussion

Findings from our analysis shows that delay in vaccination for BCG, DPT- 1st dose and Measles is high in India. Nonetheless, the median age of vaccination was within the cut-off for timely vaccination at national level as well as within all the states and union territories except for the states of Assam, Manipur and Tripura for BCG vaccination in our analysis. This suggests that the program has been moderately successful at achieving timely vaccination. There was a considerable variation in the proportion of children with delayed vaccination within states and union territories.

The proportion of children with delayed vaccination was lowest for BCG which was coherent with higher rates of institutional delivery post implementation of the National Rural Health Mission (NRHM) in 2005 [8], [9]. Compared to states with low proportion of delayed BCG vaccination the states with a high proportion of delay had lower rates of institutional delivery. DPT-1st dose, part of a multi dose schedule, is an important vaccine from the delayed vaccination perspective. Delay in the first two doses will lead to delay in the 3rd dose by default since a minimum gap of 4 weeks between 2 consecutive doses must be maintained. Similar findings have been reported earlier by studies in India and globally [19], [21], [24]. Compared to an 8-week gap between consecutive vaccine doses in high income countries like USA, the immunization schedule in India follows a 4-week gap during the first 6 months of life. This tight schedule may also lead to higher delay in vaccination for multi-dose vaccines.

Children from Muslim families had higher odds of delayed vaccination for all vaccines. Religious beliefs has an influence on the uptake of health services in general and immunization services are sensitive to these, as previous studies in India have also reported higher odds of delayed vaccination among children from Muslim families [25], [26]. Belonging to lower wealth quintiles indicates low socioeconomic status of the family and they are more likely to have lower awareness and utilisation of health services as physical, financial and social barriers to access exist, despite the provision of essential services like vaccination free of charge [19], [25], [27].

Contrary to previous studies our analysis didn’t find residing in rural area as a significant predictor for delayed vaccination for BCG, DPT-1 vaccines and for Measles vaccine, it was associated with decreased odds of delayed vaccination [19], [20], [25], [28], [29]. The primary health care system in India is better structured in rural areas post the implementation of the National Rural Health Mission and NFHS 4 is the first national level survey which captures the effect of this health system reform.

Early age at first childbirth and lower maternal education was associated with a higher odd of delayed vaccination similar to previous studies from India and other Low and middle-income countries (LMICs), as most of these women are married early and the awareness and receptiveness to health messages and uptake of health services including vaccination is lower among these women [30], [31], [32]. Maternal tetanus immunisation during pregnancy was associated with lower odds of delayed vaccination which is consistent with findings from previous NFHS data and from other studies [33], [34].

Home birth is associated with delay in seeking healthcare especially at birth and showed 2.7 times increased risk of delay in BCG as compared to births in public health facility. Several private facilities in India (especially in rural areas and small towns) provide only intra-natal care and services like vaccination may not be provided, accounting for higher odds of delayed vaccination [35], [36]

Low birth weight, especially those below 2000 g at birth and prematurity are known to be associated with vaccination delay, an important reason being parental concern about the safety and benefit of vaccination [37], [38], [39]. In our analysis children with weight <2000 g had higher odds of delayed vaccination for all vaccines, although the association was strongest for BCG, which is recommended on the first day of life.

Birth order of 4 or more was associated with delayed vaccination for Measles vaccine. This might be due to lack of time and resources for care-seeking of a non- sick child as vaccination is mainly preventive and usually accorded with a lower order of priority. This is in consensus with the earlier literature vaccination delay from India and other countries [30], [40], [41].

We didn’t find any association between female gender and delayed vaccination for BCG, DPT-1st dose and Measles in our analysis. Vaccination services are now being provided closer to home which may led greater equity in access as well as utilisation of these services. This contrasts with earlier studies which report lower utilisation of healthcare services for female child in India [24], [25], [42].

### 4.1 Strengths and limitation

The current analysis is based on the largest and most recent, nationally representative survey data available. Age calculated was done in days to ensure higher accuracy. We tried to address recall bias by restricting our analysis to children for whom date of birth and date of vaccination for a given vaccine dose were available from the MCP/Health card. Although, exclusion of children who died in this period and those without vaccination card can be a cause of potential bias and an under-estimation of delayed vaccination, a limitation of the current analysis. We couldn’t study the association of delayed vaccination with the supply side factors as the data for same isn’t captured in the NFHS-4. The cross-sectional nature of the data also precludes commenting on causal associations.

### 4.2 Recommendation/Conclusion

Vaccine timeliness is an under-recognised problem in India despite high proportion of Indian children having delayed vaccination. Timeliness of vaccination should be integral to the routine immunization programme as delayed vaccination increases the susceptibility window to vaccine preventable diseases. Targeted approach for groups with higher chances of delayed vaccination i.e. children delivered at home, low birth weight new-borns, poorer households, children of mothers with lower education, children from Muslim families may be used when designing routine immunization micro-plan in the primary care settings. Due list generated as a part of the routine immunization microplanning can be used to track the children with delay in vaccination.
