# Supplementary material for: Delayed vaccination and its predictors among children under 2 years in India: Insights from the national family health survey–4
Source: Vaccine. 2019 Apr 17;37(17):2331–9. doi: 10.1016/j.vaccine.2019.03.039 (PMC6996155; doi:10.1016/j.vaccine.2019.03.039)
Supplement: Supplementary data 1 [file mmc1.docx]

Supplementary Files

Supplementary table 1: National Immunisation Schedule for infants (children up to 1 year of age) in India.

Supplementary table 2: List of the explanatory variables and subgroups used in the analysis.

Supplementary box 1: Method of calculating age at birth and age at vaccination in days using the century day code (CDC) format

Supplementary table 1. National Immunisation Schedule for infants (children up to 1 year of age) in India.

| **Vaccine** | **Due age** | **Maximum age** |
| --- | --- | --- |
| BCG | At birth | Till 1 year of age |
| Hepatitis-B (birth dose) | At birth | Within 24 hours |
| OPV- 0 | At birth | Within first 15 days |
| OPV- 1, 2, 3 | At 6, 10 & 14 weeks | Till 5 years of age |
| Pentavalent - 1, 2, 3^#^ | At 6, 10 & 14 weeks | Till 1 year of age |
| Fractional IPV | At 6 & 14 weeks | Till 1 year of age |
| Rotavirus* | At 6, 10 & 14 weeks | Till 1 year of age |
| Pneumococcal Vaccine* | At 6 & 14 weeks (booster at 9 months) | Till 1 year of age |
| Measles / Rubella (1^st^ dose) | At 9 months-12 months | 5 years of age |
| Japanese Encephalitis –1* | At 9 months-12 months | 15 years of age |
| Vitamin A (1^st^ dose) | At 9 months | 5 years of age |

* These vaccines are currently available in selected states of the country

# (Diphtheria + Pertussis + Tetanus toxoid + Hepatitis B + Haemophilus influenza type B)

Supplementary table no 2. List of the explanatory variables and subgroups used in the analysis

| **Variable** | **Sub groups** |
| --- | --- |
| **Socio-demographic characteristics** | |
| Religion | Hindu |
|  | Muslim |
|  | Others (Christians, Sikh, Buddhist/neo-Buddhist, Jain, Jewish, Parsi/Zoroastrian, no religion, Other (not defined)) |
| Caste | Scheduled caste |
|  | Scheduled tribe |
|  | Other backward class |
|  | Others (do not belong to Scheduled caste/Tribe and other backward castes) |
| Wealth quintile | Lowest |
|  | Second |
|  | Middle |
|  | Fourth |
|  | Highest |
| Place of residence | Urban |
|  | Rural |
| **Maternal and antenatal characteristics** | |
| Maternal education | Higher (≥12 years of schooling) |
|  | Secondary (6-12 years of schooling) |
|  | Primary (≤ 5 years of schooling) |
|  | None (No schooling) |
| Maternal age at first child birth (years) | < 19 |
|  | 19 – 30 |
|  | ≥31 |
| Antenatal visits to health facility | No ANC visits |
|  | 1-3 visits |
|  | 4-7 visits |
|  | ≥8 visits |
| Financial assistance at the time of delivery | Yes |
|  | No |
| Mother had at least 1 tetanus toxoid during pregnancy with this child | Yes |
|  | No |
| **Child characteristics** | |
| Gender | Male |
|  | Female |
| Place of delivery | Home births |
|  | Private health facility |
|  | Public health facility |
| Birth weight | <2000 g |
|  | 2000-2499 g |
|  | ≥ 2500 g |
| Birth order | 1 |
|  | 2 |
|  | 3 |
|  | 4 or more |

*Sub groups were defined as per the National Family Health Survey (NFHS)-4 country report, for other subgroups the definition used has been provided

Supplementary Box 1. Method of calculating age at birth and age at vaccination in days using the century day code (CDC) format

Year of birth *365.25 = year of birth in days

Month of birth *30.45 = month of birth in days

Date of birth = date of birth in days

Age at birth (CDC) = (year of birth in days + month of birth in days +date of birth in days)

Year of vaccination for a given vaccine dose*365.25 = year of vaccination in days

Month of vaccination for a given vaccine dose*30.45 = month of vaccination in days

Date of vaccination for a given vaccine dose = date of vaccination in days

Age at vaccination for a given vaccine dose (CDC) = (year of vaccination in days + month of vaccination in days +date of vaccination in days)

Age in days at vaccination for a given vaccine dose =

**Age at vaccination for a given vaccine dose - Age at birth**
